# Consequences of sibling rivalry vary across life in a passerine bird

**DOI:** 10.1093/beheco/arw167

**Published:** 2016-12-19

**Authors:** Kat Bebbington, Sjouke A. Kingma, Eleanor A. Fairfield, Lewis G. Spurgin, Jan Komdeur, David S. Richardson

**Affiliations:** a School of Biological Sciences, University of East Anglia, Norwich Research Park, Norwich, NR4 7TJ, UK,; b Behavioural Ecology and Physiological Group, Groningen Institute for Evolutionary Life Sciences, University of Groningen, PO Box 11103, 9700CC, Groningen, The Netherlands,; c Department of Zoology, Edward Grey Institute, University of Oxford, Oxford OX1 3PS, UK, and; d Nature Seychelles, PO Box 1310, Mahé, Republic of Seychelles

**Keywords:** sibling rivalry, competition, telomere, lifetime fitness, reproductive investment, Seychelles warbler.

## Abstract

Many studies have assessed the costs of sibling rivalry in systems where offspring always have competitors, but conclusions about sibling rivalry in these species are restricted to interpreting the cost of changes in the relative level of competition and are often complicated by the expression of potentially costly rivalry related traits. Additionally, the majority of studies focus on early-life sibling rivalry, but the costs of competition can also affect later-life performance. We test a suite of hypothesized immediate (early-life body mass, telomere length, and survival) and delayed (adult reproductive potential and lifespan) costs of sibling rivalry for offspring of differing competitive ability in Seychelles warblers, where most offspring are raised singly and hence competitor success can be compared to a competition-free scenario. Compared to those raised alone, all competing nestlings had lower body mass and weaker competitors experienced reduced survival. However, the stronger competitors appeared to have longer adult breeding tenures and lifespan than those raised alone. We propose that comparisons with competition-free groups, as well as detailed fitness measures across entire lifetimes, are needed to understand the evolution of sibling rivalry and thus individual reproductive strategy in wild systems.

## Lay Summary

Offspring raised with siblings often have to compete for access to limited parental resources; but in adulthood, this prior exposure to competition may be beneficial rather than costly. In Seychelles warblers, nestlings raised with competitors suffered more than those raised alone; but during adulthood, those raised with competition appeared to outperform those raised singly. These results suggest a more multifaceted link between early-life competition and lifetime performance than is currently assumed.

## INTRODUCTION

When coexisting offspring are raised in a joint “nursery” such as in the multiple-offspring broods or litters of many vertebrates (Mock and Parker 1997), conflict between offspring for limited parental resources results in sibling rivalry (Trivers 1974; Parker et al. 2002a). Such sibling rivalry is expected to incur costs according to the degree to which the competitors’ evolutionary interests are aligned; ultimately, this depends on the direct fitness benefit of acquiring resources and the indirect fitness cost of denying them to siblings (Parker 1989).

Many studies have aimed to determine the costs of sibling rivalry for offspring (reviewed in Shaanker et al. 1988; Hudson and Trillmich 2008). When the relationship between per-capita parental investment and number of competing offspring is less than 1, offspring experience a reduction in parental resources. For each offspring, the extent of this resource-based cost depends on its relative competitive ability and the number of competitors. Although parents may have some capacity to increase overall provisioning to larger numbers of young (Hegner and Wingfield 1987), evidence for decreasing per-capita investment with increasing brood size is widespread (Mock and Forbes 1995). Reduced food intake in early life may impair a suite of physiological components (e.g., growth rates: Stamps and Tanaka 1981, body size and mass: Emlen et al. 1991, immunocompetence: Saino et al. 1997), which can in turn reduce survival to adulthood (Magrath 1991; Christe et al. 1998; Mock et al. 2009). Hence, by consuming a portion of available resources, coexisting offspring inflict a resource-based cost on each other, which may or may not be symmetrical across the brood (see below).

A second type of sibling rivalry cost concerns the behavioral adaptations that evolve as a consequence of sibling rivalry, which can be elaborate and diverse across species—ranging from nonphysical behavioral contests to obligate siblicide (Mock and Parker 1997). Sibling rivalry may be costly in terms of the production, maintenance, and expression of such traits (Godfray 1995). For example, behavioral (begging and jostling for optimal position) and physiological (growth strategies and morphological signals) adaptations to competition are found in a broad range of taxa (Manser and Avey 2000; Kilner 2001; Smiseth and Moore 2002). The energetic costs of maintaining rivalry traits, independent of parental resource depletion, may be an important component of sibling rivalry. Such traits are expected to be costly (MacNair and Parker 1979) and there is some empirical evidence for energetic costs to avian nestling begging (Kilner 2001; Neuenschwander et al. 2003). However, the magnitude of these costs appears generally limited (Smiseth and Parker 2008; reviewed in Chappell and Bachman 2002) and perhaps context-dependent (e.g., based on environmental conditions; Leech and Leonard 1996).

A third, less studied consequence of sibling rivalry is the potential for delayed costs in terms of later-life performance. If competition in early life causes suboptimal phenotypic development, it is possible that individuals become more susceptible to early mortality either through premature ageing (Nettle et al. 2015) or reduced ability to acquire resources (Merilä and Svensson 1997). Poor early-life development may also affect an individual’s ability to compete for reproduction (Verhulst et al. 1997) and this may be exacerbated if competing offspring influence the later-life reproductive potential of rivals after independence (Ekman et al. 2002; West et al. 2002; Tarwater 2012). However, very few studies have tested for such delayed costs, presumably due to the difficulty of monitoring individuals across their lifespan.

If competitive ability varies within the brood, sibling rivalry costs may be asymmetric. Competitive asymmetry typically arises through age or size differences (Mock and Forbes 1995) resulting from asynchronous birth (Drummond et al. 1986; Bonisoli-Alquati et al. 2011) or differences in growth induced by prenatal allocation of maternal resources (Einum and Fleming 1999; Royle et al. 2001). Competitor hierarchies and asymmetric competitive ability can have pronounced effects on the within-brood distribution of costs (Parker et al. 2002b), and empirical studies often suggest that the strongest competitors in a brood suffer no net cost of sibling rivalry (Cook et al. 2000; Sykes et al. 2007; Roulin and Dreiss 2012). Due to the difficulty of determining rivalry costs for the most competitive individuals (see below), the validity of this latter argument remains unclear.

Despite extensive research into sibling rivalry, there remain multiple key avenues for future research. Perhaps most importantly, many studies to date have considered broods that contain multiple offspring, where sibling rivalry will always be expected (e.g., Smale et al. 1995; Michaud and Leonard 2000, but see Emms and Verbeek 1991; Drummond et al 2011; López‐Jiménez et al. 2015).Within a brood, each individual is prenatally provisioned to deal with an expected level of competition (e.g., Harper 1986) in terms of developing the necessary morphological and behavioral platforms to express postnatal competitive traits. For individual offspring, the cost of experimentally varying the level of competition (e.g., by brood-size manipulations) will depend on the level of competition the offspring is equipped to encounter, because changing the postnatal level of competition cannot reverse the costs (or benefits) of such prenatal provisioning by parents. Thus, although previous studies have facilitated our understanding of variation in sibling rivalry, they may over or underestimate the true costs of competition, which might be better resolved by comparing competing individuals to noncompeting individuals. Importantly, a naturally occurring competition-free comparison group would best enable us to determine whether even the strongest competitors in a brood suffer rivalry costs.

In addition to the rarity of studies comparing competing and noncompeting individuals, few studies have considered competition beyond the earliest stage of dependence (but see Arroyo et al. 2002; Ekman et al. 2002; Drummond et al. 2011; Tarwater 2012). In particular, extended sibling rivalry may play an important role in social species with delayed offspring dispersal (Mock and Parker 1997); ignoring this may limit our understanding of the ultimate fitness consequences of sibling rivalry. Additionally, sibling rivalry in early life may produce delayed or ongoing costs after offspring have dispersed and no longer interact, which could affect downstream life span or reproductive performance (Spear and Nur 1994). Our knowledge about delayed sibling rivalry costs in wild systems is limited to a few studies in seabirds (Drummond et al. 2011; Müller et al. 2011; Carmona-Isunza et al. 2013)—information from a broader array of taxa is needed to infer when and how early-life rivalry has lifelong effects (Drummond et al. 2011).

The Seychelles warbler *Acrocephalus sechellensis* provides a useful system in which to improve our understanding of the lifelong costs of sibling rivalry, taking into account both prenatal priming and delayed rivalry costs outlined above. This insectivorous passerine, which is endemic to the Seychelles (Safford and Hawkins 2013), has been intensively studied on Cousin Island and provides a highly tractable system in which to explore some of the gaps in our current understanding of sibling rivalry. Modal brood size on the island is 1 but a small proportion of nests (13%) contain 2 nestlings (Komdeur 1994; Richardson et al. 2001). The fact that the majority of offspring therefore never experience competition from a coexisting nestmate and selection driving the evolution or “priming” of traits designed to manipulate competitive ability is likely to be relatively weak, we can effectively test the effect of sibling rivalry against a competition-free comparison group. Moreover, following the ca. 17-day nestling period, the Seychelles warbler has an extensive period of postfledging care (3 months, Komdeur 1991) and prolonged parent–offspring association of up to several years can occur due to habitat saturation and dispersal constraints (Komdeur 1992; Eikenaar et al. 2007),meaning that sibling rivalry can persist long after offspring become independent. Importantly, the availability of accurate reproductive and survival data allows us to test for delayed rivalry costs in terms of lifelong reproductive potential and longevity.

It is evident that there are many possible mediators and outcomes of sibling rivalry, which may have a profound influence on the evolution of reproductive strategy, resolution of evolutionary conflicts, and population dynamics. With these in mind, we test a suite of hypothesized costs of sibling rivalry (Table 1) across individuals’ entire lifetimes and determine whether these costs are greater for the weaker of 2 competitors (*asymmetric costs*, Table 1). First, we test whether nestlings with a competitor experience different *resource availability* levels to those raised alone. We then test for differences in immediate *physiological condition* as a function of rivalry in terms of early-life body mass (reflecting an individual’s energetic state; Schulte-Hostedde et al. 2005, Gil et al. 2008), and telomere length (an established predictor of intrinsic condition and survival across many taxa including the Seychelles warbler; Barrett and Richardson 2011; Barrett et al 2013). We also test for an immediate *survival cost* to rivalry in terms of survival to adulthood. Among offspring that survived to adulthood, we test the hypothesis that individuals who were raised with a competitor suffer reduced *reproductive potential* (in terms of breeding position acquisition, age at first reproduction, and breeding tenure, Table 1) and *life span*. This investigation of multiple components and consequences of sibling rivalry will enable us to disentangle the costs of competition per se and allows us to detect consequences of early-life sibling rivalry at every stage of an individual’s life span.

**Table 1:** Framework for testing hypothesized immediate and delayed costs of sibling rivalry via a suite of predictions

Fitness component	Hypothesis	Prediction	Prediction met in Seychelles warblers?	Evidence
Early life intrinsic condition and survival	*Resource availability*	Nestlings with a competitor receive less food	**Yes**—per-capita provisioning rate is lower in nests with two nestlings	Figure 1
	*Physiological condition*	a) Competing offspring have lower body mass	**Yes**—in nestlings, both A- and B-offspring have lower mass than their single counterparts	Table 2, Figure 2a
		b) Competing offspring have lower telomere length	**No**—A- and B-offspring have equal telomere length to their single counterparts	Table 2
	*Survival*	Competing offspring are less likely to survive to adulthood	**Yes**—B-offspring have lower survival than single offspring	Figure 2c, d
	*Asymmetric cost*	Physiological and recruitment costs are greater for weaker competitors	**Partially**—body mass costs apply to both competitors, survival costs only to B-offspring	Figure 2
Adult reproductive potential and survival	*Reproductive potential*	a) Competing offspring are less likely to become breeders	**No**—A- or B-offspring are equally as likely to become breeders as their single counterparts	Table 3, Figure 3a
		b) Competing offspring are slower to gain a breeding position	**No**—A- or B-offspring first breed at the same age as their single counterparts	Table 3, Figure 3b
		c) Competing offspring have shorter breeding tenures	**No**—A-offspring have longer breeding tenures than their single counterparts	Table 3, Figure 3c
	*Life span*	Competing offspring have lower lifespans	**No**—A-offspring have longer lifespans than their single counterparts	Table 3, Figure 3d
	*Asymmetric cost*	Reproductive potential and lifespan costs are greater for weaker competitors	**No**—B-offspring have similar reproductive potential and lifespan to their single counterparts	Table 3, Figure 3

## METHODS

### Study system and field data

Data were collected in the Seychelles warbler population on Cousin Island between 1995 and 2014. Across this period of intensive study, nearly all birds on the island received a unique British Trust for Ornithology ring and a combination of color rings for individual identification (Richardson et al 2001; Hammers et al. 2013). Each year during the main breeding season (June–September) and in some years during the minor breeding season (January–March; Komdeur 1996), a census of the entire population was conducted followed by intense monitoring of all nesting attempts on the island. These censuses, combined with negligible off-island dispersal (Komdeur et al. 2004), yield a >90% re-sighting probability (Brouwer et al. 2006) so death dates can be accurately inferred from the time of disappearance from the population. Each season, the majority of first-year birds were caught and ringed either as nestlings (ca. Day 10 of the nestling period during a small window of development within which nestlings are big enough to fit with rings but small enough not to present a risk of force-fledging), dependent fledglings, or independent subordinates in their natal territory. Age at catch was determined by eye color (Komdeur 1991); in this study we only use data from birds caught when <1 year of age and distinguish between dependent (fledglings observed begging, <3 months, gray eyes) and independent (3–11 months, brown eyes) individuals. To determine *physiological condition*, body mass (to 0.1 g) and tarsus length (to 0.1 mm) were recorded and a small blood sample (ca. 25 µl) was taken via brachial venipuncture and stored in absolute ethanol.

Seychelles warblers defend year-round territories occupied by a breeding pair and 0–5 independent subordinates (Komdeur 1992). The identity of the breeding pair in each territory was determined from behavioral interactions during censuses (Richardson et al. 2003). Nesting attempts were located by following the breeding female for signs of nesting activity. If the nest was accessible (by hand or using a pole and mirror), the clutch and/or brood size was recorded. All nests were followed until failure or fledging (hatching and fledgling success are 46% and 80%, respectively [Komdeur 1994]). In a small proportion of nests, partial brood loss may mean that one nestling died before the brood size was recorded. To minimize error in our brood size classification, we therefore only classified nestlings as “single” if they were alone in the nest on or before Day 12 of the nestling period. However, we were able to record the clutch and hatching brood size for 41% of nestlings and the remaining 59% were, on average, classified earlier than Day 12 (mean ± SE = 8 ± 4 days). Thus, although some “single” nestlings may therefore have had a nestmate that died prior to the classification, the proportion is likely to be small (we were only aware of 3 partially fledged nests in our nestling dataset). Furthermore, the direction of any error will be in the opposite direction to the hypotheses in Table 1, thus making our assignment conservative. To determine survival to adulthood for all sampled nestlings, fledglings, and independent offspring, we recorded the presence of each individual in the population in the year following birth and all surviving individuals were subsequently followed for their entire lives as part of continued seasonal monitoring to determine adult reproduction and life span (Table 1).

In order to test for *asymmetric costs* (Table 1), we calculated each nestling’s body condition as the residuals of a regression of mass on tarsus length, controlling for the time of day and month in which sampling took place, separately for males and females. Where 2 nestlings from the same brood were sampled, we used body condition to determine each offspring’s size rank and assigned them as either the A-offspring (higher condition) or B-offspring (lower condition). Ranking competitors in this way reduces the variance in condition in each group compared to that of single offspring; in order to make a more meaningful comparison with our competition-free comparison group, we therefore also assigned each single nestling either as a “high-quality” or “low-quality” single offspring according to whether its body condition fell above or below the mean condition of all single offspring. A-offspring and B-offspring could then be compared to similarly classified single counterparts rather than to all single offspring.

The Seychelles warbler has obligate biparental care (Komdeur 1992) and subordinates can become helpers-at-the-nest by incubating or provisioning nestlings—the latter increases total provisioning rate to the brood (Komdeur 1994, Richardson et al. 2002). For 86 nests, food provisioning watches of approximately 1 hour (mean duration ± SD = 64.3 ± 13.2) were conducted on Days 10–11 of the nestling period (mean age ± SD = 10.7 ± 5.1) to quantify overall nest provisioning rate (the number of provisioning events per hour) and to determine which (if any) subordinates helped in provisioning. Watches were focused around this stage of the nestling period to coincide with approximate asymptote of provisioning rate. For a small subset of nests (*n* = 20), a provisioning watch was also conducted on Day 3 of the nestling period. We used this subset of nests to determine the repeatability of our provisioning rate measures (see Statistical methods). We tested the *resource availability* hypothesis (Table 1) by calculating per-capita provisioning rate as the total provisioning rate divided by brood size. Observations of nestling provisioning provide evidence that food partitioning is equal between nestlings (Supplementary Appendix B, see Discussion for details).

There is pronounced spatial and temporal variation in habitat quality on Cousin (Brouwer et al. 2006). During each season, the quality of every territory was calculated as a function of foliage density, insect abundance, and territory size following Komdeur (1992) and Brouwer et al. (2006). In this study, we define territory quality as the natural log of this measure and per-capita territory quality as territory quality divided by the number of independent birds (>3 months) present in the territory that season, following Brouwer et al. (2006). Insect availability across the island also varies annually, so for each season we calculated food availability as the mean number of insects counted across the whole island during each breeding season following Brouwer et al. (2006).

### Molecular methods

DNA for molecular sexing and telomere measurement was extracted using a DNeasy blood and tissue kit (Qiagen) according to the manufacturer’s instructions with modification of overnight lysis at 37 °C and a final DNA elution volume of 80 μL. We determined the sex of all offspring using the PCR method developed by Griffiths et al. (1998).

We used quantitative PCR (qPCR) to obtain relative telomere length (henceforth telomere length) measurements as described for the Seychelles warbler in full detail elsewhere (Barrett et al. 2013; Bebbington et al. 2016). Briefly, we ran each DNA sample in duplicate and used LinRegPCR 2014.2 to correct baseline fluorescence, determine the window-of-linearity for each amplicon, and calculate individual well efficiencies. Threshold values (Nq) were set in the center of the window-of-linearity per amplicon for all samples. We corrected for variation across plates using a golden sample inter-plate calibrator and then calculated telomere length for each sample as the amount of telomere DNA relative to that of a constantly expressed reference gene (GAPDH) that was simultaneously amplified on the same plate, following equation 1 in Pfaffl (2001).

### Statistical analyses

We examined the costs of sibling rivalry using a total of 349 nestling and juvenile Seychelles warblers. Unless stated otherwise, all analyses were conducted using a mixed modeling procedure in the lme4 (Bates et al. 2015) package in R (R core team 2015). All models included year of birth to account for variation in island density, climate and resources across years. In models using data from two individuals from the same nest we also included nest identity to account for nonindependence between nestmates. We removed variables for which *P* > 0.05 from the final reported models. Stepwise elimination of nonsignificant variables can increase the likelihood of type I error (Mundry and Nunn 2009), but can be appropriate in cases of specific hypothesis testing with a small number of variables (Bolker et al. 2009), as is the case in this study. We minimize the potential for type I error by reintroducing all excluded variables back into the minimum model before considering them nonsignificant (*P* > 0.05 in all combinations). We report estimates from the final model including only significant terms and fixed effects; we obtained estimates for nonsignificant terms by reintroducing these terms individually to the final minimum adequate model.

To test for differences in *resource availability*, we first tested for inherent differences in the physical and social environment between nests containing 1 and 2 nestlings. We modelled brood size as a binomial response and tested for relationships with territory quality, food availability, and group size. In our investigation of variation in per-capita provisioning rate, we first determined how well per-capita provisioning rate reflects general resource availability at a given nest. Using the 20 nests for which a Day 3 provisioning watch was also performed, we built a linear model with Day 10 provisioning rate as the response variable and tested the strength of relationship with Day 3 provisioning rate. Using each nest as a single data point, we then examined whether per-capita provisioning rate on Day 10 (response variable) was related to brood size. We included 1) brood size, 2) helper presence (only 9 [5%] nests had >1 helper), 3) nest age in days, 4) observation time (early: 0630–1100; mid: 1100–1500; late: 1500–1800 hours), because provisioning rate may vary across the day (e.g., Knapton 1984), 5) territory quality, and 6) food availability, as provisioning rate may depend on resource availability or foraging time (e.g., Tremblay et al. 2005). These latter 2 measures are correlated (*R*^2^ = 0.17), but not strongly enough to cause colinearity in our analysis (VIF = 1.08). We also tested whether helper presence, territory quality, and food availability interacted with brood size.

We examined *physiological condition* separately in nestlings and juveniles by testing the relationship between size rank and 2 Gaussian response variables: body mass and telomere length. In nestlings, we created separate models for high-quality (A-offspring and high-quality single offspring) and low-quality (B-offspring and low-quality single offspring) categories. In juveniles, we compared all A-, B-, and single offspring together to maximize power under limited sample sizes.

We tested whether body mass was related to competitor presence and size rank. We included time (classified as above) and month of capture, the interaction between tarsus length and sex (to account for sex-specific scaling of mass and tarsus), territory quality, and food availability (which may affect offspring body mass through maternal effects [Richardson et al. 2004, Russell et al. 2007] or provisioning rate to offspring [Schroeder et al. 2012]) as additional predictors. For nestlings, we also included helper presence to account for varying food acquisition and for juveniles we included sampling age (dependent or independent) and used the per-capita measure of territory quality to account for group-size mediated postfledging competition (Brouwer et al 2006; Ridley and Raihani 2007). To investigate telomere length, we used the same additional predictors as for body mass. For nestlings, we also added tarsus length to control for variation in growth rates between nestlings. In all models, we tested for interactions between competitor presence or size rank and food availability and territory quality; and in nestlings, we also tested the interaction with helper presence.

To analyze *survival* to adulthood of nestlings and juveniles, we used a generalized linear mixed model with a binomial error structure and survival to adulthood as a binary response. In nestlings, we performed the quality-based comparisons described above: A-offspring versus higher-quality single offspring and B-offspring versus lower-quality single offspring. In juveniles, we compared all A-, B-, and single offspring. We did not include food availability or territory quality based on a prior study reporting no effect of these variables on juvenile survival (Brouwer et al. 2006).

Among individuals that survived to adulthood, we compared the *reproductive potential* and *life span* of A- and B-offspring with that of their single counterparts as described above. Some individuals in our dataset (*n* = 19) were selected at random to be translocated to different islands as part of a planned expansion of the species’ range (Richardson et al. 2006; Wright et al. 2014)—any of these individuals that did not yet hold a breeding position when translocated were excluded from our analyses of breeding position acquisition and age at first reproduction and all translocated individuals were excluded from analyses of breeding tenure and life span. Acquisition of a breeding position was modeled as a binomial response in a standard generalized linear model, excluding 3 individuals who were still alive at the time of analysis but had not yet gained a breeding position (2 single offspring and 1 B-offspring). We investigated age at first reproduction, breeding tenure, and life span using cox proportional hazards survival analyses in the “survival” package (Therneau 2015) in R. Because some individuals were still alive at the time of analysis, our data were left-censored: each individual was classified as either dead or alive in the model. The assumption of proportional hazards were met in all models (Cox 1972). We report the hazard coefficient, or “risk”, of becoming a breeder (age at first reproduction), ceasing to be a breeder (breeding tenure), and dying (life span) for individuals who had a competitor compared to those raised alone, separately for high- and low-quality offspring. We included sex and group size (number of independent birds in the territory) as additional predictors in all models to account for potential sex differences in breeding performance and group-size–mediated differences in reproductive opportunities. We also tested the interactions between these 2 predictors and competitor presence.

## RESULTS

Our nestling dataset contained 161 (71%) single nestlings and 66 (29%) nestlings with a nestmate. For simplicity, we report model estimates for size rank and any additional predictors of early-life sibling rivalry costs for which *P* < 0.25. Model estimates for all other nonsignificant additional predictors and nonsignificant interaction terms are available in Supplementary Appendix A.

### Resource availability in nestlings

Brood size was not significantly related to territory quality (β ± SE = −0.30 ± 0.21, *P* = 0.15) or food availability (β ± SE = 0.01 ± 0.01, *P* = 0.51), but did increase with group size (β ± SE = 0.36 ± 0.14, *P* = 0.01).

**Figure 1:**
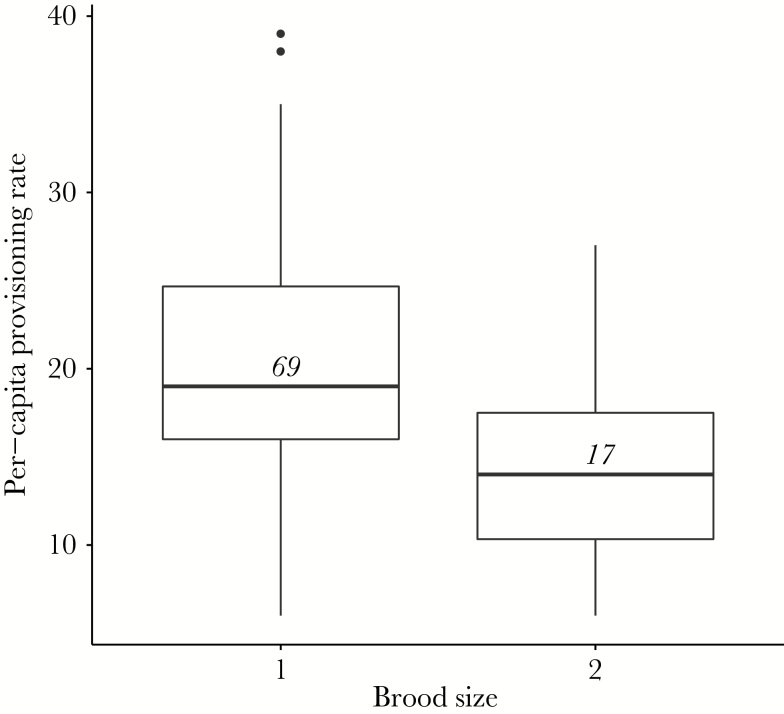
Boxplot showing median (horizontal line) per-capita provisioning rate to nestlings with and without a competitor. Numbers on each box denote sample sizes per group. Nestlings with a competitor received significantly less food than those raised alone (β ± SE = −5.76 ± 1.79, *P* = 0.002).

Among nests where 2 provisioning watches were conducted, the per-capita provisioning rates of the 2 watches were significantly positively correlated (β ± SE = 0.55 ± 0.14, *P* < 0.01) with an R-squared of 0.45 (Supplementary Figure 1). This repeatability suggests that our Day 10 measures of per-capita provisioning rate reflect general resource availability at a given nest. Across all nests for which we had Day 10 provisioning data (*n* = 86), nestlings with a nestmate each received less food than those raised alone (Figure 1) as found in a previous study (Komdeur 1994). Per-capita provisioning rate varied throughout the day ([β ± SE vs. morning: afternoon 1.01 ± 1.74, *P* = 0.56; evening 4.21 ± 1.71, *P* = 0.02). There was a nonsignificant tendency for per-capita provisioning rate to increase with helper presence [β ± SE = 2.46 ± 1.48, *P* = 0.10] but neither food availability nor territory quality affected per-capita provisioning rate and there were no significant interactions between brood size and any other variables (Supplementary Table 1).

### Physiological condition

**Figure 2:**
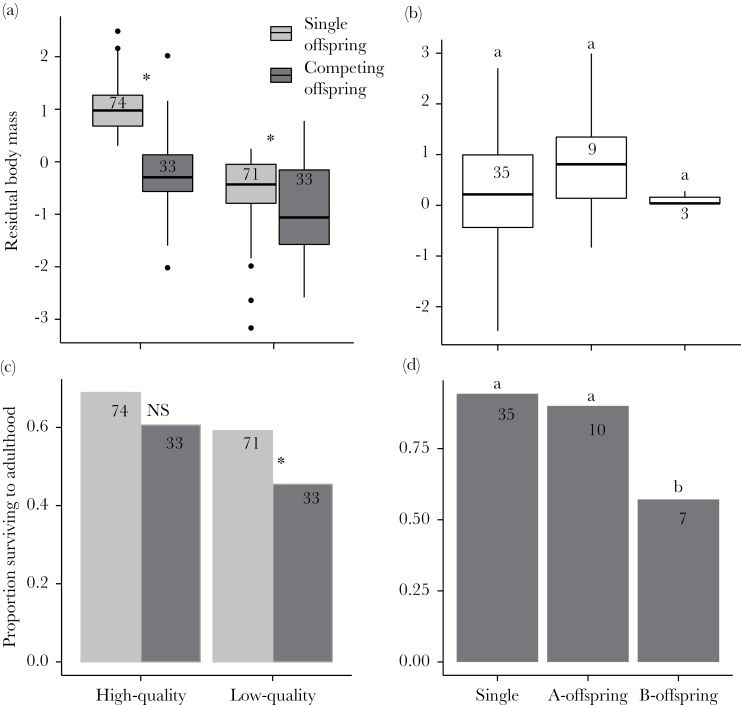
Early life body condition and recruitment costs of sibling rivalry. a) Nestling body condition; b) Juvenile body condition; c) Nestling survival to adulthood; d) Juvenile survival to adulthood. In nestlings, high-quality refers to A-offspring and single offspring with greater than average body condition, and low-quality refers to B-offspring and single offspring with lower than average body condition (see Methods). * = significant relationships, NS = non-significant relationships. In juveniles, A- and B-offspring are compared with all single offspring. Different letters between groups denote significant differences. Throughout, numbers denote sample sizes per group, boxplots display median values per group, and bar plots display mean values per group.

In nestlings, the body mass of both A- and B-offspring was lower than that of their single counterparts (Figure 2a, Table 2). Territory quality, food availability, and helper presence had no effect on nestling mass and were not significant in interactions with size rank (Supplementary Table 2). Nestling telomere length did not vary with size rank (Table 2) but declined with increasing tarsus length in low-quality individuals, likely as a function of increasing nestling age (Table 2). Food availability, territory quality, and helper presence had no effect on nestling telomere length and did not significantly interact with size rank (Supplementary Table 2).

**Table 2:** Predictors of nestling and juvenile body mass and telomere length in Seychelles warblers

Physiological measure	Comparison	Predictor	Estimate ± SE	*P*-value
Nestling body mass (*n* = 211)	High-quality^1^	**Competitor presence**	**−1.23 ± 0.14**	**<0.01**
		**Catch time (vs. morning**)	**Mid 0.31 ± 0.15**	**0.04**
			**Late 0.44 ± 0.17**	**0.01**
		**Tarsus length × Sex**	**0.20 ± 0.07**	**<0.01**
	Low-quality	**Competitor presence**	**−0.52 ± 0.18**	**<0.01**
		Catch time (vs. morning)	Mid 0.29 ± 0.18	0.11
			Late 0.27 ± 0.23	0.24
		**Catch month**	**0.17 ± 0.06**	**<0.01**
		Tarsus length × Sex	0.13 ± 0.09	0.18
Nestling telomere length (*n* = 172)	High-quality	Tarsus length	−0.03 ± 0.02	0.12
		Competitor presence	−0.05 ± 0.09	0.60
	Low-quality	**Tarsus length**	**−0.06 ± 0.03**	**0.02**
		Competitor presence	−0.08 ± 0.10	0.43
Juvenile body mass (*n* = 46)	All offspring	Age (vs. independent)	−1.07 ± 0.58	0.07
		Size rank	A-offspring 0.24 ± 0.48	0.62
			B-offspring −0.16 ± 0.57	0.78
Juvenile telomere length (*n* = 44)	All offspring	Size rank	A-offspring −0.10 ± 0.08	0.21
			B-offspring 0.13 ± 0.10	0.22

Significant terms are in bold. ^1^In nestlings, we tested for physiological costs for A- and B-offspring separately with respect to their single offspring counterparts (see main text). High-quality refers to A-offspring and single offspring where body condition > single offspring mean, and low-quality refers to B-offspring and single offspring where body condition < single offspring mean.

Juvenile body mass was not related to nestling size rank (Figure 2b, Table 2) but the sample size for B-offspring was very low. None of the additional predictors were related to juvenile body mass (Supplementary Table 2), nor were present in interactions (Supplementary Table 2). Juvenile telomere length was not related to size rank (Table 2) nor to any additional predictors (Supplementary Table 2) and there was no interaction between size rank and any other predictor on juvenile telomere length (Supplementary Table 2).

### Survival cost

In nestlings, there was not a significant difference between the survival of A-offspring and their single counterparts (β ± SE = −0.47 ± 0.47, *P* = 0.32, Figure 2c) but B-offspring were significantly less likely to survive to adulthood than low-quality single offspring (β ± SE = −1.00 ± 0.50, *P* = 0.04 Figure 2c). A similar pattern occurred in juveniles: A-offspring were equally likely to survive as single offspring (Figure 2d), but B-offspring were less likely to survive than single offspring (β ± SE = −2.80 ± 1.09, *P* = 0.01, Figure 2d). B-offspring tended to have lower survival than A-offspring, but not significantly so (β ± SE = −2.20 ± 1.33, *P* = 0.10, Figure 2d).

### Reproductive potential and life span

**Figure 3:**
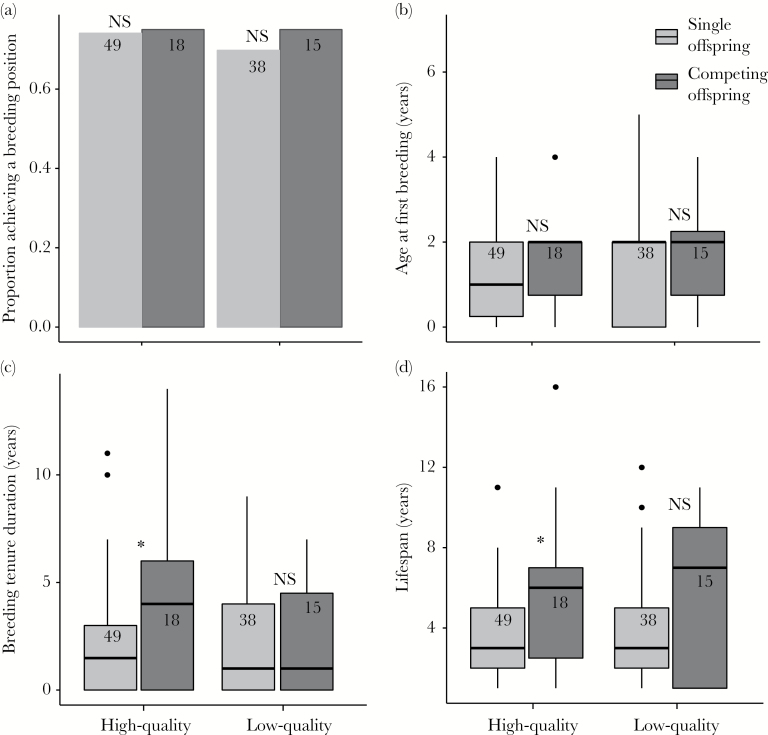
The relationship between competitor presence and a) proportion of individuals acquiring a breeding position, b) age at which the breeding position was attained, c) length of the breeding tenure, and d) adult lifespan among individuals surviving to adulthood. High- and low-quality groups are defined as for Figure 2a (see Methods). * = significant relationships, NS = nonsignificant relationships. Throughout, numbers denote sample sizes per group, boxplots display median values per group, and bar plots display mean values per group.

Among individuals that survived to adulthood, neither competitor presence (Figure 3a) nor group size influenced the likelihood of achieving a breeding position either for high-quality or low-quality offspring (Table 3), although males in the high-quality category were slightly more likely to become breeders (*P* = 0.08). Competitor presence (Figure 3b), group size, and sex were also unrelated to age at first reproduction in both high- and low-quality offspring (Table 3). A-offspring had longer breeding tenures than their singleton counterparts, as indicated by a lower hazard ratio (Table 3), but the breeding tenure of B-offspring did not differ from low-quality single offspring (Table 3, Figure 3c). Among both low- and high-quality offspring, individuals from larger groups had lower breeding tenures, as indicated by a higher hazard ratio (Table 3). A-offspring also had longer life spans than their single counterparts, whereas the life span of B-offspring and low-quality single offspring did not differ (Table 3, Figure 3d). In both high-and low-quality categories, individuals from larger groups had lower life spans, as indicated by a positive hazard ratio (Table 3). There were no interactions between competition and either sex or group size for any of the 3 reproductive components or life span for either high- or low-quality offspring (Supplementary Table 3).

**Table 3:** Predictors of *reproductive potential* and *life span* among Seychelles warbler offspring that survived to adulthood

Reproductive component	Comparison	Predictor	Coefficient ± SE	*P*	Hazard ratio
Achieved breeding status (*n* = 104)	High-quality	Competing offspring	0.52 ± 0.73	0.48	
		Group size	−0.27 ± 0.38	0.47	
		Sex (male)	1.23 ± 0.71	0.08	
	Low-quality	Competing offspring	−0.32 ± 0.73	0.67	
		Group size	−0.49 ± 0.31	0.12	
		Sex (male)	−0.68 ± 0.71	0.34	
Age at first reproduction (*n* = 102)	High-quality	Competitor presence	−0.22 ± 0.29	0.44	0.80
		Group size	0.10 ± 0.18	0.56	1.11
		Sex (male)	0.04 ± 0.27	0.87	1.04
	Low-quality	Competitor presence	−0.52 ± 0.36	0.15	0.59
		Group size	0.22 ± 0.13	0.08	1.26
		Sex (male)	0.48 ± 0.32	0.13	1.61
Breeding tenure (*n* = 100)	High-quality	**Competitor presence**	**−0.82 ± 0.37**	**0.03**	**0.44**
		**Group size**	**0.65 ± 0.21**	**<0.01**	**1.92**
		Sex (male)	−0.37 ± 0.32	0.25	0.69
	Low-quality	Competitor presence	−0.37 ± 0.39	0.34	0.69
		**Group size**	**0.47 ± 0.16**	**<0.01**	**1.60**
		Sex	0.24 ± 0.34	0.49	1.28
Lifespan (*n* = 100)	High-quality	**Competitor presence**	**−0.76 ± 0.36**	**0.04**	**0.47**
		**Group size**	**0.58 ± 0.21**	**<0.01**	**1.78**
		Sex	−0.12 ± 0.27	0.67	0.89
	Low-quality	Competitor presence	−0.49 ± 0.40	0.21	0.61
		**Group size**	**0.43 ± 0.15**	**<0.01**	**1.53**
		Sex	0.43 ± 0.35	0.22	1.54

The analysis of whether individuals achieved breeding status was performed with a logistic regression: all other models were based on survival analyses. Hazard ratio describes the risk of the event (becoming a breeder, ceasing to be a breeder or dying) for an individual raised with a competitor relative to an individual raised alone, such that values below 1 indicate less risk to competing individuals. Significant terms are in bold.

## DISCUSSION

In this study, we tested a suite of hypothesized mediators and costs of sibling rivalry (Table 1). We found evidence for decreasing *resource availability* as a function of increased brood size, which translated into reduced *physiological condition* in both A- and B-nestlings when compared to competition-free, single nestlings of the same quality category. However, the *survival* cost imposed by having a competitor was asymmetric within broods: in nestlings, only B-offspring had lower survival than their single counterparts, and in juveniles, B-offspring were less likely to survive than single offspring. Among individuals who survived to adulthood, the relationship between sibling rivalry and adult *reproductive potential* and *life span* was positive for A-offspring, who outperformed their single counterparts in terms of breeding tenure and life span, and neutral for B-offspring, who performed equally well as their single counterparts in all tested aspects of adult success. We discuss these results in detail below.

### Universal immediate costs: resource availability and physiological condition

Individuals in larger broods may suffer from resource depletion as a function of the number or strength of competitors (Forbes et al. 1997; Kitaysky et al. 2001), which can lead to reduced body condition (Emlen et al. 1991) and recruitment rates (Schwagmeyer and Mock 2008). In our dataset, we found no evidence that brood size was linked to territory quality or food availability, suggesting that resource depletion as a function of increased brood size is not mitigated by increased overall resource availability. We also found that nestlings with a competitor received substantially less food than those raised alone. This suggests that the reduced body mass found in competing nestlings is, at least partly, the result of reduced food intake; but without quantifying nestling begging behavior, we cannot rule out additional energetic costs of behavioral competition. However, evidence for energetic costs of begging is limited (e.g., McCarty 1996; Chappell and Bachman 2002) and we suspect that such costs are low in the Seychelles warbler. Intra-brood scramble competition (Stamps et al. 1978; MacNair and Parker 1979) should occur whenever parents allocate nondivisible resources among nestlings (Royle et al 1999), but anecdotal observations by the authors suggest that Seychelles warbler parents usually bring multiple small insects to the nest in a given trip and divide them equally between the nestlings (pers. obs). Preliminary evidence collected earlier in the Seychelles warbler long-term study also shows that provisioning rate to each nestling appears approximately equal (Supplementary Table 4); although we acknowledge that we do not have sufficient data for a formal statistical analysis, taken together this anecdotal evidence is compatible with the hypothesis that resource-based rivalry costs should be relatively equal between the 2 competitors. The fact that A-offspring have lower nestling body mass than the highest-quality single offspring (Figure 2a) suggests that A-offspring do indeed suffer a cost associated with the presence of the B-offspring, but whether or not the relative extent of this cost is greater for B-offspring is difficult to determine. Differences in juvenile body mass and telomere length between A- and B-offspring would have allowed us to better determine whether *physiological condition* does indeed differ between competitors, but we found no differences in telomere length according to size rank. This lack of any effect may be due to the low power of our tests involving telomere measures, given the number of individuals involved (*n* = 172 nestlings and 44 juveniles). It may also be because telomeres lack the resolution to reflect differences in condition at the scale at which it was considered here. It would be interesting to test for differences in other physiological characteristics, such as immune function, between A- and B-offspring to determine whether either, or both, competitors suffer with respect to *physiological condition* more generally.

### Asymmetric immediate costs: survival to adulthood

Although *physiological condition* was reduced among nestling competitors regardless of size rank, only B-offspring had lower nestling survival to adulthood than their single counterparts. In juveniles, B-offspring also experienced lower survival than all single offspring (Table 2) and tended to have lower survival than A-offspring, although this last result was not significant (*P* = 0.10).Together these results suggests that the *physiological costs* of sibling rivalry in early life have a disproportionately large impact on the survival of weaker competitors. If we apply the brood reduction (where weak offspring only survive in favorable circumstances [O’Connor 1978]) and egg insurance (where extra offspring are produced to mitigate the potential loss of a more valuable “core” offspring [Mock and Forbes 1995]) hypotheses to the Seychelles warbler system, we would predict that second eggs constitute a bet-hedging strategy by parents to optimize their reproductive output. We believe this to be unlikely for several reasons. First, B-offspring fledge as often as those raised alone (in all but three of the nests in the nestling analysis, the entire brood fledged) and we found no interaction between food availability and competitive ability on offspring condition (Supplementary Table 2). Second, approximately half of all nests containing 2 nestlings are the result of communal breeding of 2 females (Richardson et al. 2001) and it seems unlikely that this breeding strategy would remain stable if 1 female was restricted to laying an insurance egg (e.g., Clutton-Brock 1998). Third, environmental predictability is very high in this system (Komdeur and Pels 2005) and so selection for “parental optimism” (Mock and Forbes 1995) in relation to brood size is likely to be weak. We therefore suggest that variation in brood size in this species is likely to reflect variation in parental perception of the likelihood of success of the whole brood.

### Asymmetric delayed costs: adult reproductive potential and life span

Although our results clearly support the *physiological condition* and *survival* hypotheses of sibling rivalry in early life, we found limited support for the *reproductive potential* hypothesis. In contrast to our predictions, A-offspring who survived to adulthood had longer breeding tenures than high-quality single offspring and also lived longer than their single counterparts. Additionally, B-offspring had equal breeding tenure and survival to their single counterparts, so do not seem to be suffering any later-life costs to sibling rivalry if they survive to adulthood. A lack of later-life cost for B-offspring has also been shown in blue-footed boobies *Sula nebouxii*, where B-offspring suffer neither reduced survival nor reduced immunocompetence in adulthood (Drummond et al. 2011; Carmona-Isunza et al. 2013). These results suggest that, provided they reach adulthood, B-offspring are able to buffer any negative effects of early-life stress (Drummond et al. 2003).

However, the positive effect of sibling rivalry on A-offspring adult performance is perhaps more perplexing. As Seychelles warblers typically occupy a breeding position until death (Hammers et al. 2015), breeding tenure and life span are inherently linked and we suggest that the positive effect of rivalry on A-offspring adult performance could arise through 3 nonmutually exclusive mechanisms. First, A-offspring may outperform single offspring because broods of 2 are only produced under highly favorable circumstances. Our results show that this is not the case in terms of territory quality or food availability, but it is possible that A-offspring are sired by better-quality parents and thus inherit that quality. However, because nestling body mass of A-offspring is lower than that of higher-quality single offspring, this seems an unlikely explanation. Second, it is possible that A-offspring who survive to adulthood are of higher quality or competitive ability due to some selective filter on poor-quality individuals, which leads to biases either in death rates or in tendency for individuals to gain a breeding position (as oppose to remaining as a subordinate in a territory). Finally, A-offspring may become better competitors through exposure to competition early in life and are therefore better able to obtain a higher-quality breeding position, where the costs of obtaining food and producing offspring are relatively low. Once in the breeding territory, low costs could result in greater somatic maintenance and hence life span. Empirical evidence, although rare, suggests that such early-life influence on behavioral phenotype can occur: in yellow-legged gull chicks *Larus michahellis*, last-hatch nestlings produce very different behavioral responses to first-hatch nestlings (Diaz-Real et al. 2016) and in Nazca boobies *Sula granti*, nestlings that experience more adult aggression tend to be more aggressive later in life (Müller et al. 2011). Due to the correlation inherent to individual resource availability and intrinsic condition, it is difficult to distinguish between these 2 latter alternatives. However, given that A-offspring do not out-perform single offspring during the first year of life, it at least seems likely that any observed “benefits” of competition for A-offspring arise after independence, either as a result of selective mortality or competitive traits that are not expressed until adulthood. We suggest that investigating behavioral and social competence as a function of early-life competition would be a highly interesting avenue for further study.

### Sibling rivalry costs and competition-free comparisons

Parents can optimize the level of sibling rivalry to maximize their own fitness by creating asymmetric competitive hierarchies. These can arise through asynchronous hatching of eggs (Ricklefs 1993) or preferential allocation of pre or postnatal resources to specific offspring (Slagsvold 1997; Groothuis et al. 2005). Many studies of sibling rivalry have shown that costs are often much greater for weaker siblings as a result of these hierarchies (e.g., Mock and Ploger 1987; Forbes and Glassey 2000; Smiseth et al. 2007). However, studies often fail to determine the costs of competition per se, as many systems do not provide the opportunity to compare competing and noncompeting offspring. The costs for dominant siblings may therefore be masked by the level of rivalry expected in the population and the costs for weaker offspring underestimated. Our comparison between nestlings that were raised with and without competition did not involve experimental manipulations, hence we are unable to rule out all potential parental or environmental factors that might differ between these 2 groups. Nonetheless, our results suggest that comparison between competing and noncompeting offspring, experimentally assigned where possible, can provide important insights and enhance our understanding of sibling rivalry costs. For example, if the current study had compared 2-chick nests with nests containing 3 chicks (as are found on other isolated islands in the Seychelles warbler’s range [Komdeur et al. 1995]), we may have concluded that the physiological costs of sibling rivalry only affected second- or third-order nestlings. It was only through comparison with single offspring and specifically single offspring of a similar quality category, that we were able to detect an absolute cost of competition. Similarly, by removing single offspring from our analysis of juvenile *recruitment*, we may have concluded that there was no recruitment cost to rivalry, whereas actually B-offspring suffered relative to single offspring. These results add further support to the hypothesis of asymmetric costs of competition within broods, but also suggest a need to consider more global costs and benefits within families in order to understand the multiple drivers and mediators of sibling rivalry and reproductive strategy. However, it is important to note that the correlational nature of the current study limits our ability to control for variation in parental quality, which may influence the degree to which offspring raised with and without rivalry differ. Given that per-capita provisioning rate is lower in broods of 2, it seems reasonable to assume that nestlings raised with a competitor experience some kind of resource limitation regardless of any differences in parental quality; nonetheless, studies that are able to experimentally separate the effects of parental quality and sibling rivalry are required to more comprehensively explore the extend of sibling rivalry costs.

### CONCLUSIONS

In this study, we used a comprehensive framework of hypothesized costs to understand the manifestation and extent of sibling rivalry in wild systems. Although our results provide strong evidence for both asymmetrical and universal costs of sibling rivalry, we also found that stronger competitors that did overcome the early-life costs of rivalry had a longer breeding tenure and life span than single offspring. We suggest that comparisons of individuals raised with and without sibling competition, combined with detailed monitoring of individuals throughout life, will be instrumental in future studies of sibling rivalry, evolution of parental investment, and individual reproductive strategies in wild systems.

## SUPPLEMENTARY MATERIAL

Supplementary material can be found at http://www.beheco.oxfordjournals.org/.

## FUNDING

This work was supported by 2 Natural Environment Research Council grants to D.S.R. (NE/F02083X/1 and NE/K005502/1) on which J.K. was a project partner.

## Supplementary Material

Online_appendices

## References

[CIT0001] Arroyo BE de Cornulier T Bretagnolle V 2002 Parental investment and parent–offspring conflicts during the postfledging period in Montagu’s harriers. Anim Behav. 63:235–244.

[CIT0002] Barrett EL Richardson DS 2011 Sex differences in telomeres and lifespan. Aging Cell. 10:913–921. 21902801 10.1111/j.1474-9726.2011.00741.x

[CIT0003] Barrett EL Burke TA Hammers M Komdeur J Richardson DS 2013 Telomere length and dynamics predict mortality in a wild longitudinal study. Mol Ecol. 22:249–259. 23167566 10.1111/mec.12110

[CIT0004] Bates D Maechler M Bolker B Walker S 2015 Fitting linear mixed-effects models using lme4. J Stat Softw. 67:1–48.

[CIT0005] Bebbington K Spurgin LG Fairfield EA Dugdale HL Komdeur J Burke T Richardson DS 2016 Telomere length reveals cumulative individual and transgenerational inbreeding effects in a passerine bird. Mol Ecol. 25:2949–2960. 27184206 10.1111/mec.13670 PMC4999029

[CIT0006] Bebbington K 2016 Data from: consequences of sibling rivalry vary across life in a passerine bird. Dryad Digital Repository. http://dx.doi.org/10.5061/dryad.12np0. 10.1093/beheco/arw167 PMC5873840 29622918

[CIT0007] Bolker BM Brooks ME Clark CJ Geange SW Poulsen JR Stevens MH White JS 2009 Generalized linear mixed models: a practical guide for ecology and evolution. Trends Ecol Evol. 24:127–135. 19185386 10.1016/j.tree.2008.10.008

[CIT0008] Bonisoli-Alquati A Boncoraglio G Caprioli M Saino N 2011 Birth order, individual sex and sex of competitors determine the outcome of conflict among siblings over parental care. Proc Biol Sci. 278:1273–1279. 20943688 10.1098/rspb.2010.1741 PMC3049075

[CIT0009] Brouwer L Richardson DS Eikenaar C Komdeur J 2006 The role of group size and environmental factors on survival in a cooperatively breeding tropical passerine. J Anim Ecol. 75:1321–1329. 17032364 10.1111/j.1365-2656.2006.01155.x

[CIT0010] Carmona-Isunza MC Núñez-de la Mora A Drummond H 2013 Chronic stress in infancy fails to affect body size and immune response of female boobies or their offspring. J. Avian. Biol. 44: 390–398.

[CIT0011] Chappell MA Bachman GC 2002 Energetic costs of begging behavior. In: Wright J Leonard ML, editors. The evolution of begging: competition, cooperation, and communication. Dordrecht (the Netherlands): Kluwer Adademic Publishers p. 43–162.

[CIT0012] Christe P Møller AP de Lope F 1998 Immunocompetence and nestling survival in the house martin: the tasty chick hypothesis. Oikos. 83:175–179.

[CIT0013] Clutton-Brock TH 1998 Reproductive skew, concessions and limited control. Trends Ecol Evol. 13:288–292. 21238306 10.1016/s0169-5347(98)01402-5

[CIT0014] Cook MI Monaghan P Burns MD 2000 Effects of short-term hunger and competitive asymmetry on facultative aggression in nestling black guillemots *Cepphusgrylle*. Behav Ecol. 11:282–287.

[CIT0015] Cox DR 1972 Regression models and life tables (with discussion). J. R. Statist. Soc. B. 34: 187–220.

[CIT0016] Diaz-Real J Kim SY Velando A 2016 Hatching hierarchy but not egg-related effects governs behavioral phenotypes in gull chicks. Behav Ecol. 27: 1782–1789.

[CIT0017] Drummond H González E Osorno JL 1986 Parent-offspring cooperation in the blue-footed boody (*Sula nebouxii*): social roles in infanticial brood reduction. Behav Ecol Sociobiol. 19:365–372.

[CIT0018] Drummond H Torres R Krishnan VV 2003 Buffered development: resilience after aggressive subordination in infancy. Am Nat. 161:794–807. 12858285 10.1086/375170

[CIT0019] Drummond H Rodríguez C Oro D 2011 Natural ‘poor start’ does not increase mortality over the lifetime. Proc Biol Sci. 278:3421–3427. 21450729 10.1098/rspb.2010.2569 PMC3177622

[CIT0020] Eikenaar C Richardson DS Brouwer L Komdeur J 2007 Parent presence, delayed dispersal, and territory acquisition in the Seychelles warbler. Behav Ecol. 18:874–879.

[CIT0021] Einum S Fleming IA 1999 Maternal effects of egg size in brown trout (*Salmo trutta*): norms of reaction to environmental quality. Proc Biol Sci. 266:2095–2100.

[CIT0022] Ekman J Eggers S Griesser M 2002 Fighting to stay: the role of sibling rivalry for delayed dispersal. Anim Behav. 64:453–459.

[CIT0023] Emlen ST Wrege PH Demong NJ Hegner RE 1991 Flexible growth rates in nestling white-fronted bee-eaters: a possible adaptation to short-term food shortage. Condor. 93:591–597.

[CIT0024] Emms SK Verbeek NA 1991 Brood size, food provisioning and chick growth in the Pigeon Guillemot *Cepphus columba*. Condor. 93:943–951.

[CIT0025] Forbes S Thornton S Glassey B Forbes M Buckley NJ 1997 Why parent birds play favourites. Nature. 390: 351–352.

[CIT0026] Forbes S Glassey B 2000 Asymmetric sibling rivalry and nestling growth in red-winged blackbirds (*Agelaius phoeniceus*). Behav Ecol Sociobiol. 48:413–417.

[CIT0027] Gil D Bulmer E Celis P López-Rull I 2008 Adaptive developmental plasticity in growing nestlings: sibling competition induces differential gape growth. Proc Biol Sci. 275:549–554. 18089540 10.1098/rspb.2007.1360 PMC2596805

[CIT0028] Godfray HCJ 1995 Signaling of need between parents and young: parent-offspring conflict and sibling rivalry. Am Nat. 146:1–24.

[CIT0029] Griffiths R Double MC Orr K Dawson RJ 1998 A DNA test to sex most birds. Mol Ecol. 7:1071–1075. 9711866 10.1046/j.1365-294x.1998.00389.x

[CIT0030] Groothuis TG Müller W von Engelhardt N Carere C Eising C 2005 Maternal hormones as a tool to adjust offspring phenotype in avian species. Neurosci Biobehav Rev. 29:329–352. 15811503 10.1016/j.neubiorev.2004.12.002

[CIT0031] Hammers M Richardson DS Burke T Komdeur J 2013 The impact of reproductive investment and early-life environmental conditions on senescence: support for the disposable soma hypothesis. J Evol Biol. 26:1999–2007. 23961923 10.1111/jeb.12204

[CIT0032] Hammers M Kingma SA Bebbington K van de Crommenacker J Spurgin LG Richardson DS Burke T Dugdale HL Komdeur J 2015 Senescence in the wild: Insights from a long-term study on Seychelles warblers. Exp Gerontol. 71:69–79. 26344178 10.1016/j.exger.2015.08.019

[CIT0033] Harper AB 1986 The evolution of begging: sibling competition and parent-offspring conflict. Am Nat. 128:99–114.

[CIT0034] Hegner RE Wingfield JC 1987 Effects of brood-size manipulations on parental investment, breeding success, and reproductive endocrinology of house sparrows. Auk. 104:470–80.

[CIT0035] Hudson R Trillmich F 2008 Sibling competition and cooperation in mammals: challenges, developments and prospects. Behav Ecol Sociobiol. 62:299–307.

[CIT0036] Kilner RM 2001 A growth cost of begging in captive canary chicks. Proc Natl Acad Sci USA. 98:11394–11398. 11572988 10.1073/pnas.191221798 PMC58740

[CIT0037] Kitaysky AS Wingfield JC Piatt JF 2001 Corticosterone facilitates begging and affects resource allocation in the black-legged kittiwake. Behav Ecol. 12:619–625.

[CIT0038] Knapton RW 1984 Parental feeding of nestling Nashville Warblers: the effects of food type, brood-size, nestling age, and time of day. Wilson Bull. 96:594–602.

[CIT0039] Komdeur J 1991 Cooperative breeding in the Seychelles warbler [PhD thesis]. [UK]: University of Cambridge.

[CIT0040] Komdeur J 1992 Importance of habitat saturation and territory quality for the evolution of cooperative breeding in the Seychelles warbler. Nature. 358:493–495.

[CIT0041] Komdeur J 1994 Experimental evidence for helping and hindering by previous offspring in the cooperative-breeding Seychelles warbler *Acrocephalus sechellensis*. Behav Ecol Sociobiol. 34:175–186.

[CIT0042] Komdeur J 1996 Seasonal timing of reproduction in a tropical bird, the Seychelles warbler: a field experiment using translocation. J Biol Rhythms. 11:333–346. 8946261 10.1177/074873049601100407

[CIT0043] Komdeur J Pels MD 2005 Rescue of the Seychelles warbler on Cousin Island, Seychelles: the role of habitat restoration. Biol Conserv. 124:15–26.

[CIT0044] Komdeur J Huffstadt A Prast W Castle G Mileto R Wattel J 1995 Transfer experiments of Seychelles warblers to new islands: changes in dispersal and helping behavior. Anim Behav. 49:695–708.

[CIT0045] Komdeur J Piersma T Kraaijeveld K Kraaijeveld‐Smit F Richardson DS 2004 Why Seychelles warblers fail to recolonize nearby islands: unwilling or unable to fly there? Ibis. 146:298–302.

[CIT0046] Leech SM Leonard ML 1996 Is there an energetic cost to begging in nestling tree swallows (Tachycineta bicolor)? Proc Biol Sci. 263:983–987.

[CIT0047] López‐Jiménez L Blas J Tanferna A Cabezas S Marchant T Hiraldo F Sergio F 2015 Ambient temperature, body condition and sibling rivalry explain feather corticosterone levels in developing black kites. Funct Ecol. 30:605–613.

[CIT0049] Macnair MR Parker GA 1979 Models of parent-offspring conflict III: Intrabrood conflict. Anim Behav. 27:1202–1209.

[CIT0050] Magrath RD 1991 Nestling weight and juvenile survival in the blackbird, *Turdus merula*. J Anim Ecol. 60:335–351.

[CIT0051] Manser MB Avey G 2000 The effect of pup vocalisations on food allocation in a cooperative mammal, the meerkat (*Suricata suricatta*).Behav Ecol Sociobiol. 48:429–437.

[CIT0052] McCarty JP 1996 The energetic cost of begging in nestling passerines. Auk. 1:178–188.

[CIT0053] Merilä J Svensson E 1997 Are fat reserves in migratory birds affected by condition in early life? J Avian Biol. 28:279–286.

[CIT0054] Michaud T Leonard M 2000 The role of development, parental behavior, and nestmate competition in fledging of nestling tree swallows. Auk. 117:996–1002.

[CIT0055] Mock DW Ploger BJ 1987 Parental manipulation of optimal hatch asynchrony in cattle egrets: an experimental study. Anim Behav. 35:150–60.

[CIT0056] Mock DW Parker GA 1997 The evolution of sibling rivalry. Oxford: Oxford University Press.

[CIT0057] Mock DW Forbes LS 1995 The evolution of parental optimism. Trends Ecol Evol. 10:130–134. 21236982 10.1016/s0169-5347(00)89014-x

[CIT0058] Mock DW Schwagmeyer PL Dugas MB 2009 Parental provisioning and nestling mortality in house sparrows. Anim Behav. 78: 677–684.

[CIT0059] Müller MS Porter ET Grace JK Awkerman JA Birchler KT Gunderson AR Schneider EG Westbrock MA Anderson DJ 2011 Maltreated nestlings exhibit correlated maltreatment as adults: evidence of a “cycle of violence” in Nazca boobies (*Sula granti*). Auk. 128:615–619.

[CIT0060] Mundry R Nunn CL 2009 Stepwise model fitting and statistical inference: turning noise into signal pollution. Am Nat. 173:119–123. 19049440 10.1086/593303

[CIT0061] Nettle D Monaghan P Gillespie R Brilot B Bedford T Bateson M 2015 An experimental demonstration that early-life competitive disadvantage accelerates telomere loss. Proc Biol Sci. 282:20141610. 25411450 10.1098/rspb.2014.1610 PMC4262165

[CIT0062] Neuenschwander S Brinkhof MW Kölliker M Richner H 2003 Brood size, sibling competition, and the cost of begging in great tits (*Parus major*). Behav Ecol. 14:457–462.

[CIT0063] O’Connor RJ 1978 Brood reduction in birds, selection for infanticide, fratricide, and suicide? Anim Behav. 26:79–96.

[CIT0064] Parker GA 1989 Hamilton’s rule and conditionality. EtholEcolEvol. 1: 195–211.

[CIT0065] Parker GA Royle NJ Hartley IR 2002a. Intrafamilial conflict and parental investment: a synthesis. Philos Trans R Soc Lond B Biol Sci. 357:295–307. 11958698 10.1098/rstb.2001.0950 PMC1692944

[CIT0066] Parker GA Royle NJ Hartley IR 2002b. Begging scrambles with unequal chicks: interactions between need and competitive ability. Ecol Lett. 5:206–215.

[CIT0067] Pfaffl MW 2001 A new mathematical model for relative quantification in real-time RT-PCR. Nucleic Acids Res. 29:e45. 11328886 10.1093/nar/29.9.e45 PMC55695

[CIT0068] R Core Team. 2015 R: a language and environment for statistical computing. Vienna (Austria): R Foundation for Statistical Computing.

[CIT0069] Richardson DS Burke T Komdeur J 2002 Direct benefits and the evolution of female-biased cooperative breeding in Seychelles warblers. Evolution. 56:2313–2321. 12487360 10.1111/j.0014-3820.2002.tb00154.x

[CIT0070] Richardson DS Bristol R Shah NJ 2006 Translocation of the Seychelles warbler *Acrocephalus sechellensis* to establish a new population on Denis Island, Seychelles. Conserv Evidence. 3:54–57.

[CIT0071] Richardson DS Jury FL Blaakmeer K Komdeur J Burke T 2001 Parentage assignment and extra-group paternity in a cooperative breeder: the Seychelles warbler (*Acrocephalus sechellensis*). Mol Ecol. 10:2263–2273. 11555268 10.1046/j.0962-1083.2001.01355.x

[CIT0072] Richardson DS Komdeur J Burke T 2004 Inbreeding in the Seychelles warbler: environment-dependent maternal effects. Evolution. 58:2037–2048. 15521460 10.1111/j.0014-3820.2004.tb00488.x

[CIT0073] Richardson DS Burke T Komdeurs J 2003 Sex-specific associative learning cues and inclusive fitness benefits in the Seychelles warbler. J Evol Biol. 16:854–861. 14635900 10.1046/j.1420-9101.2003.00592.x

[CIT0074] Ricklefs RE 1993 Sibling competition, hatching asynchrony, incubation period, and lifespan in altricial birds. In: Power DM, editor. Current Ornithology. Vol. 11 New York: Plenum Press p. 199–276.

[CIT0075] Ridley AR Raihani NJ 2007 Variable postfledging care in a cooperative bird: causes and consequences. Behav Ecol. 18:994–1000.

[CIT0076] Roulin A Dreiss AN 2012 Sibling competition and cooperation over parental care. In: Royle NJ Smiseth PT Kölliker M, editors. The evolution of parental care. Oxford, UK: Oxford University Press p. 133–149.

[CIT0077] Royle NJ Hartley IR Owens IP Parker GA 1999 Sibling competition and the evolution of growth rates in birds. Proc Biol Sci. 266:923–932.

[CIT0078] Royle NJ Surai PF Hartley IR 2001 Maternally derived androgens and antioxidants in bird eggs: complementary but opposing effects? Behav Ecol. 12:381–385.

[CIT0079] Russell AF Langmore NE Cockburn A Astheimer LB Kilner RM 2007 Reduced egg investment can conceal helper effects in cooperatively breeding birds. Science. 317:941–944. 17702942 10.1126/science.1146037

[CIT0080] Safford R Hawkins F 2013 The Seychelles warbler. In: The birds of Africa: Volume VII: the Malagasy region: Madagascar, Seychelles, Comoros, Mascarenes. London: Christopher Helm p. 758–7670.

[CIT0081] Saino N Calza S Moller AP 1997 Immunocompetence of nestling barn swallows in relation to brood size and parental effort. J Anim Ecol. 66:827–836.

[CIT0082] Schroeder J Nakagawa S Cleasby IR Burke T 2012 Passerine birds breeding under chronic noise experience reduced fitness. PLoS One. 7:e39200. 22808028 10.1371/journal.pone.0039200 PMC3394753

[CIT0083] Schulte-Hostedde AI Zinner B Millar JS Hickling GJ 2005 Restitution of mass-size residuals: validating body condition indices. Ecology. 86:155–163.

[CIT0084] Schwagmeyer PL Mock DW 2008 Parental provisioning and offspring fitness: size matters. Anim. Behav. 75: 291–298.

[CIT0085] Shaanker RU Ganeshaiah KN Bawa KS 1988 Parent-offspring conflict, sibling rivalry, and brood size patterns in plants. Annu Rev Ecol Syst. 19:177–205.

[CIT0086] Slagsvold T 1997 Brood division in birds in relation to offspring size: sibling rivalry and parental control. Anim Behav. 54:1357–1368. 9521793 10.1006/anbe.1997.0530

[CIT0087] Smale L Holekamp KE Weldele M Frank LG Glickman SE 1995 Competition and cooperation between litter-mates in the spotted hyaena, *Crocuta crocuta*. Anim Behav. 50:671–682.

[CIT0088] Smiseth PT Moore AJ 2002 Does resource availability affect offspring begging and parental provisioning in a partially begging species? Anim Behav. 63:577–585.

[CIT0089] Smiseth PT Ward RJ Moore AJ 2007 Parents influence asymmetric sibling competition: experimental evidence with partially dependent young. Ecology. 88:3174–3182. 18229851 10.1890/06-1992.1

[CIT0090] Smiseth PT Parker HJ 2008 Is there a cost to larval begging in the burying beetle Nicrophorusvespilloides? Behav Ecol. 19:1111–1115.

[CIT0091] Spear LB Nur N 1994 Brood size, hatching order and hatching date: effects on four life history stages from hatching to recruitment in western gulls. J Anim Ecol. 63:283–298.

[CIT0092] Stamps J Tanaka S 1981 The influence of food and water on growth rates in a tropical lizard (*Anolis aeneus*). Ecology. 62:33–40.

[CIT0093] Stamps J Metcalf RA Krishnan VV 1978 A genetic analysis of parent-offspring conﬂict. Behav Ecol Sociobiol. 3:369–392.

[CIT0094] Sykes EM Innocent TM Pen I Shuker DM West SA 2007 Asymmetric larval competition in the parasitoid wasp *Nasonia vitripennis*: a role in sex allocation? Behav Ecol Sociobiol. 61:1751–1758.

[CIT0095] Tarwater CE 2012 Influence of phenotypic and social traits on dispersal in a family living, tropical bird. Behav Ecol. 23:1242–1249.

[CIT0096] Therneau T 2015 A package for survival analysis in S. Version 2.38. Available from: http://CRAN.R-project.org/package=survival.

[CIT0097] Tremblay I Thomas D Blondel J Perret P Lambrechts MM 2005 The effect of habitat quality on foraging patterns, provisioning rate and nestling growth in Corsican Blue Tits *Parus caeruleus*. Ibis. 147:17–24.

[CIT0098] Trivers RL 1974 Parent-offspring conflict. Am Zool. 14:249–264.

[CIT0099] Verhulst S Perrins CM Riddington R 1997 Natal dispersal of great tits in a patchy environment. Ecology. 78:864–872.

[CIT0100] West SA Pen I Griffin AS 2002 Cooperation and competition between relatives. Science. 296:72–75. 11935015 10.1126/science.1065507

[CIT0101] Wright DJ Spurgin LG Collar NJ Komdeur J Burke T Richardson DS 2014 The impact of translocations on neutral and functional genetic diversity within and among populations of the Seychelles warbler. Mol Ecol. 23:2165–2177. 24689851 10.1111/mec.12740 PMC4237152

