# Supplementary material for: Consequences of sibling rivalry vary across life in a passerine bird
Source: Behav Ecol. 2016 Dec 19;28(2):407–18. doi: 10.1093/beheco/arw167 (PMC5873840; doi:10.1093/beheco/arw167)
Supplement: Online_appendices [file arw167_suppl_online_appendices.docx]

Supporting information for “Consequences of sibling rivalry vary across life in a passerine bird”

**Supplementary Appendix A: Additional model outputs for analyses of sibling rivalry**

**Table S1** Predictors of per-capita provisioning rate in Seychelles warbler nests (n = 86). Significant terms are in bold and estimates of main effects are reported from a linear model without interactions.

| Predictor | Estimate ± SE | CI | | *P* |
| --- | --- | --- | --- | --- |
|  |  | Lower | Upper |  |
| **Brood size** | **-5.76± 1.79** | **-9.21** | **-2.30** | **0.002** |
| **Observation time (vs early)** | Mid 1.01 ± 1.74  **Late 4.21 ± 1.71** | -2.37  **0.90** | 4.33  **7.51** | 0.56  **0.02** |
| Helper presence | 2.46 ± 1.48 | -0.38 | 5.31 | 0.10 |
| Annual food availability | 0.06 ± 0.05 | -0.03 | 0.16 | 0.25 |
| Nest age | 0.19 ± 0.18 | -0.15 | 0.53 | 0.30 |
| Territory quality | 0.67 ± 1.09 | -1.44 | 2.72 | 0.54 |
| Brood size * food availability | 0.18 ± 0.11 | -0.03 | 0.39 | 0.11 |
| Brood size * territory quality | 1.97 ± 2.41 | -2.82 | 6.33 | 0.42 |
| Brood size * helper presence | -1.16 ± 3.72 | -8.27 | 5.96 | 0.76 |

**Table S2** Model estimates for nonsignificant terms and interactions between competitive status and other explanatory variables of nestling and juvenile body condition and RTL.

| Physiological measure | Comparison | Predictor | Estimate ± SE | *P* |
| --- | --- | --- | --- | --- |
| Nestling body mass  (*n* = 211) | High-quality | Annual food availability | <-0.01 ± <0.01 | 0.49 |
|  |  | Helper presence | -0.05 ± 0.15 | 0.73 |
|  |  | Territory quality | <0.01 ± 0.10 | 0.95 |
|  |  | Catch month | 0.06 ± 0.07 | 0.37 |
|  |  | Competitor presence * helper presence | 0.18 ± 0.30 | 0.55 |
|  |  | Competitor presence * food availability | <-0.01 ± 0.01 | 0.68 |
|  |  | Competitor presence * territory quality | -0.05 ± 0.20 | 0.81 |
|  | Low-quality | Annual food availability | <-0.01 ± <0.01 | 0.44 |
|  |  | Helper presence | -0.14 ± 0.19 | 0.47 |
|  |  | Territory quality | 0.03 ± 0.13 | 0.81 |
|  |  | Competitor presence * helper presence | -0.35 ± 0.24 | 0.14 |
|  |  | Competitor presence * food availability | - 1. 0.01 | 0.44 |
|  |  | Competitor presence * territory quality | 0.18 ± 0.27 | 0.51 |
| Nestling RTL  (*n* = 172) | High-quality | Helper presence | -0.09 ± 0.08 | 0.27 |
|  |  | Annual food availability | <-0.01 ± <0.01 | 0.40 |
|  |  | Territory quality | - 1. 0.07 | 0.60 |
|  |  | Competitor presence * territory quality | <-0.01 ± 0.13 | 0.99 |
|  |  | Competitor presence * helper presence | <0.01 ± 0.17 | 0.99 |
|  |  | Competitor presence * food availability | <-0.01 ± <0.01 | 0.55 |
|  | Low-quality | Annual food availability | <-0.01 ± <0.01 | 0.72 |
|  |  | Territory quality | -0.02 ± 0.07 | 0.76 |
|  |  | Helper presence | -0.03 ± 0.10 | 0.77 |
|  |  | Competitor presence * helper presence | -0.20 ± 0.20 | 0.31 |
|  |  | Competitor presence * food availability | <-0.01 ± <0.01 | 0.38 |
|  |  | Competitor presence * territory quality | -0.04 ± 0.15 | 0.78 |
| Juvenile  body mass  (*n* = 46) | All offspring | Annual food availability | 0.03 ± 0.03 | 0.38 |
|  |  | Per-capita territory quality | <-0.01 ± 0.25 | 0.98 |
|  |  | Catch time (versus morning) | Mid -0.08 ± 0.41  Late 0.48 ± 0.52 | 0.85  0.36 |
|  |  | Catch month | 0.04 ± 0.07 | 0.59 |
|  |  | Tarsus length * sex | -0.52 ± 0.31 | 0.31 |
|  |  | Competitor presence * food availability | A-offspring 0.02 ± 0.11  B-offspring 0.13 ± 1.39 | 0.87  0.92 |
|  |  | Competitor presence * territory quality | A-offspring -1.37 ± 0.61  B-offspring -2.36 ± 5.26 | 0.09  0.67 |
| Juvenile RTL (*n* = 44) | All offspring | Annual food availability | <0.01 ± <0.01 | 0.25 |
|  |  | Per-capita territory quality | 0.05 ± 0.06 | 0.40 |
|  |  | Age (vs independent) | 0.02 ± 0.10 | 0.85 |
|  |  | Competitor presence * food availability | A-offspring 0.02 ± 0.02  B-offspring 0.17 ± 0.23 | 0.31  0.47 |
|  |  | Competitor presence * territory quality | A-offspring <0.01 ±0.16  B-offspring -0.49 ± 1.08 | 0.97  0.65 |

Table S3 Model estimates for nonsignificant interactions between predictors of reproductive performance and competitive status among individuals that survived to adulthood.

| Reproductive component | Comparison | Predictor | Estimate ± SE | *P* | Hazard ratio |
| --- | --- | --- | --- | --- | --- |
| Achieved breeding status  (n = 104) | High-quality | Competitor presence * group size | -1.35 ± 1.02 | 0.18 |  |
|  |  | Competitor presence * sex | -3.32 ± 1.85 | 0.06 |  |
|  | Low-quality | Competitor presence * group size | -0.22 ± 0.72 | 0.76 |  |
|  |  | Competitor presence * sex | -0.07 ± 1.58 | 0.97 |  |
| Age at first reproduction  (n = 102) | High-quality | Competitor presence * group size | -0.14 ± 0.39 | 0.72 | 0.87 |
|  |  | Competitor presence * sex | 0.21 ± 0.58 | 0.72 | 1.23 |
|  | Low-quality | Competitor presence * group size | 0.27 ± 0.29 | 0.38 | 1.29 |
|  |  | Competitor presence * sex | 0.97 ± 0.87 | 0.27 | 2.63 |
| Breeding tenure  (n = 100) | High-quality | Competitor presence * group size | -0.04 ± 0.40 | 0.92 | 0.96 |
|  |  | Competitor presence * sex | -0.14 ± 0.72 | 0.85 | 0.87 |
|  | Low-quality | Competitor presence * group size | 0.37 ± 0.36 | 0.30 | 1.44 |
|  |  | Competitor presence * sex | 0.85 ± 0.83 | 0.31 | 2.33 |
| Lifespan  (n = 100) | High-quality | Competitor presence * group size | -0.11 ± 0.36 | 0.76 | 0.90 |
|  |  | Competitor presence * sex | -0.05 ± 0.60 | 0.93 | 0.60 |
|  | Low-quality | Competitor presence * group size | 0.16 ± 0.30 | 0.60 | 1.17 |
|  |  | Competitor presence * sex | 0.86 ± 0.77 | 0.26 | 2.36 |

**Supplementary Appendix B: Division of food items between competing nestlings**

**Table S4** Division of parental provisioning between nestlings in two-chick nests, observed between 1987 and 1990. Provisioning watches were performed for one hour on ca day 10 of the nestling period. The provisioning rate to each nesting was calculated as the number of times that that individual received food. The least-fed offspring was classified as nestling 1.

| Nest number | Nest provisioning rate (Feeds/h) | Nestling 1 | | Nestling 2 | |
| --- | --- | --- | --- | --- | --- |
|  |  | Provisioning rate | Proportion of feeds | Provisioning rate | Proportion of feeds |
| 1 | 15 | 6.3 | 0.42 | 8.7 | 0.58 |
| 2 | 22 | 9 | 0.41 | 13 | 0.59 |
| 3 | 22.8 | 8.9 | 0.39 | 13.9 | 0.61 |
| 4 | 18 | 9 | 0.5 | 9 | 0.5 |
| 5 | 33.9 | 16.2 | 0.48 | 17.7 | 0.52 |
| 6 | 16.1 | 7.2 | 0.45 | 8.9 | 0.55 |
| **Average** |  |  | **0.44** |  | **0.56** |
